# Brain Iron Deposits in Thalamus Is an Independent Factor for Depressive Symptoms Based on Quantitative Susceptibility Mapping in an Older Adults Community Population

**DOI:** 10.3389/fpsyt.2019.00734

**Published:** 2019-10-15

**Authors:** Wenhua Zhang, Ying Zhou, Qingqing Li, Jinjin Xu, Shenqiang Yan, Jinsong Cai, Yeerfan Jiaerken, Min Lou

**Affiliations:** ^1^Department of Neurology, the Second Affiliated Hospital of Zhejiang University, School of Medicine, Hangzhou, China; ^2^Department of Radiology, the Second Affiliated Hospital of Zhejiang University, School of Medicine, Hangzhou, China

**Keywords:** iron deposits, white matter hyperintensities, depressive symptoms, quantitative susceptibility mapping, thalamus

## Abstract

**Objectives:** With the trend of an aging population, an increasing prevalence of late-life depression has been identified. Several studies demonstrated that iron deposition was significantly related to the severity of symptoms in patients with depression. However, whether brain iron deposits influence depressive symptoms is so far unclear in the community of older adults. We measured iron deposition in deep intracranial nucleus by quantitative susceptibility mapping (QSM) and aimed to explore the relationship between iron deposition and depressive symptoms.

**Methods:** We reviewed the data of a community population from CIRCLE study, which is a single-center prospective observational study that enrolled individuals above 40 years old with cerebral small vessel disease (SVD), while free of known dementia or stroke. We evaluated regional iron deposits on QSM, measured the volume of white matter hyperintensities (WMHs) on T2 fluid-attenuated inversion recovery, and assessed depressive symptoms by Hamilton depression scale (HDRS). We defined depressive symptom as HDRS > 7.

**Results:** A total of 185 participants were enrolled. Participants in depressive symptom group had higher QSM value in thalamus than control group (18.79 ± 14.94 vs 13.29 ± 7.64, *p* = 0.003). The QSM value in the thalamus was an independent factor for the presence of depressive symptoms (OR = 1.055; 95% CI: 1.011-1.100; p = 0.013). The regional QSM values in other areas were not associated with HDRS score (all p > 0.05). No significant correlations were observed between WMHs volume and HDRS score (p > 0.05), or regional QSM values and WMHs volume (all p > 0.05).

**Conclusions:** Our study demonstrated that iron deposits in the thalamus were related to the depressive symptoms in older adults.

## Introduction

With the trend of an aging population, an increasing prevalence of late-life depression has been identified. The devastating effects of depression in older adults have been reported, including increases in suicide, hastened cognitive decline, worsening physical comorbidities, higher caregiver burden and all-cause mortality (1, 2). However, the mechanism of depression in older adults remains uncertain.

As age increases, intracranial iron deposits increase (3, 4). Studies have indicated that increased iron deposits in the deep gray matter of the brain are closely related to neurodegenerative diseases, such as Parkinson’s disease (PD) and Alzheimer’s disease (AD) (5). Recent studies also show that increased intracranial iron deposits are associated with emotional behaviors among PD patients, especially depression (6). Moreover, Yao et al. (7) also demonstrated that patients with major depression disorder had a significantly increased susceptibility value in the bilateral putamen than patients with mild-moderate depression or control subjects. Therefore, we presume that in older adults, brain iron deposits might be related to depressive symptoms.

White matter hyperintensities (WMHs), demonstrating related to ischemic, inflammatory and protein deposition, which are commonly seen as confluent or patchy hyperintense areas on T2 weighted or fluid-attenuated inversion recovery (FLAIR) scans, have been reported to be related to both iron deposition and depression in older adults (8). However, the relationship between iron deposits and WMHs is still controversial. Yan et al. (9) observed a significant association between iron deposits in globus pallidus and WMHs volume among patients admitted to hospital, while other study revealed that intracranial iron deposition was not associated with the volume of WMHs (10). Besides, numerous studies have demonstrated that there is a significant but weak association between WMHs and depression (11). However, the relationship between iron deposits, WMHs and depression in older adults remains uncertain.

In previous studies, R2* was usually used to measure iron content, which was easily affected by different factors, such as calcification. The current study has suggested that quantitative susceptibility mapping (QSM) measures iron deposits more accurately (12). Moreover, postmortem study also found that QSM values were directly proportional to iron content (13). Therefore, in this study we evaluated iron deposition in deep intracranial nucleus on QSM, measured the volume of WMHs on T2-FLAIR, and assessed depressive symptoms using the Hamilton depression scale (HDRS), with the aim to explore the relationship between iron deposition and depressive symptoms, and the role of WMHs among them.

## Materials and Methods

### Subjects

The CIRCLE study (ClinicalTrials.gov ID: NCT03542734) was a single-center prospective observational study that enrolled community residents, which aimed to explore the predictors of small vessel disease (SVD) and cognitive deficits. We reviewed the data of consecutive individuals from CIRCLE cohort between 2017 October and 2018 July. Detailed inclusion criteria was: (1) age above 40; (2) SVD imaging markers (WMHs with Fazekas score 1-3 in periventricular or deep white matter, lacunes, microbleeds) visible on MRI; (3) free of known dementia or stroke (both cerebral infarction and hemorrhage); (4) without any MRI contraindications; (5) free of serious head injury (resulting in the loss of consciousness) or received intracranial surgery; (6) not suffering from cancer. Participants with poor image quality due to motion artifact or with the history of psychotropic drugs were excluded. All participants received neuropsychological testing, retinal digital images and multimodal MRI.

### MRI Protocol

All subjects underwent a multi-model MRI by a 3.0 T MR (HDXT, GE Healthcare, United States) scanner using an 8- channel brain phased array coil, including T1, T2 fluid-attenuated inversion recovery (FLAIR), and susceptibility weighted imaging (SWI) sequence. In order to minimize head motion, foam pads were inserted into the space between the subject’s head and the MRI head coil. An axial T2 FLAIR sequence was used to measure the WMHs volume with the following parameters: repetition time = 8000 ms, echo time = 150 ms, FOV = 24 cm × 24 cm, matrix size = 256 × 256, inversion time = 2100 ms, slice thickness = 4.0 mm with no gap (continuous) between slices, and in-plane spatial resolution of 0.4688 mm/pixel × 0.4688 mm/pixel. The whole brain was imaged. The SWI sequence was in an axial orientation parallel to the anterior commissure to posterior commissure line and covered the whole lateral ventricles, using a three-dimension multi-echo gradient-echo sequence with 11 equally spaced echoes: echo time = 4.5 ms [first echo], inter-echo spacing = 4.5 ms, repetition time = 34 ms, FOV = 24 cm × 24 cm, matrix size = 416 × 384, slice thickness = 2.0 mm with no gap between slices, and in-plane spatial resolution of 0.93 mm/pixel × 0.93 mm/pixel. Flow compensation was applied.

### Volume Assessment of WMHS

First, the axial T2 FLAIR images were segmented automatically through the lesion segmentation tool (LST) in MATLAB (R2014a) pipeline integrating SPM12 (Wellcome Department of Neurology, University College of London, UK). Then the automatically segmented lesions were manually checked and corrected on mricron (http://www.nitrc.org/projects/mricron) by two experienced neuro-radiologists (WZ and YJ) who were blinded to all other imaging and clinical data after it was coregistered to the T1 images through SPM12. The manual correction process included: (1) division of deep white matter hyperintensities (DWMHs) and periventricular white matter hyperintensities (PVHs); (2) correction of non-white matter area being labeled as WMHs; (3) WMHs area not adequately labeled as WMHs or normal-appearing white matter falsely labeled as WMHs. Afterwards, the volume of WMHs was measured automatically on mricron.

### Measurement of the QSM Values

**Figure 1:**
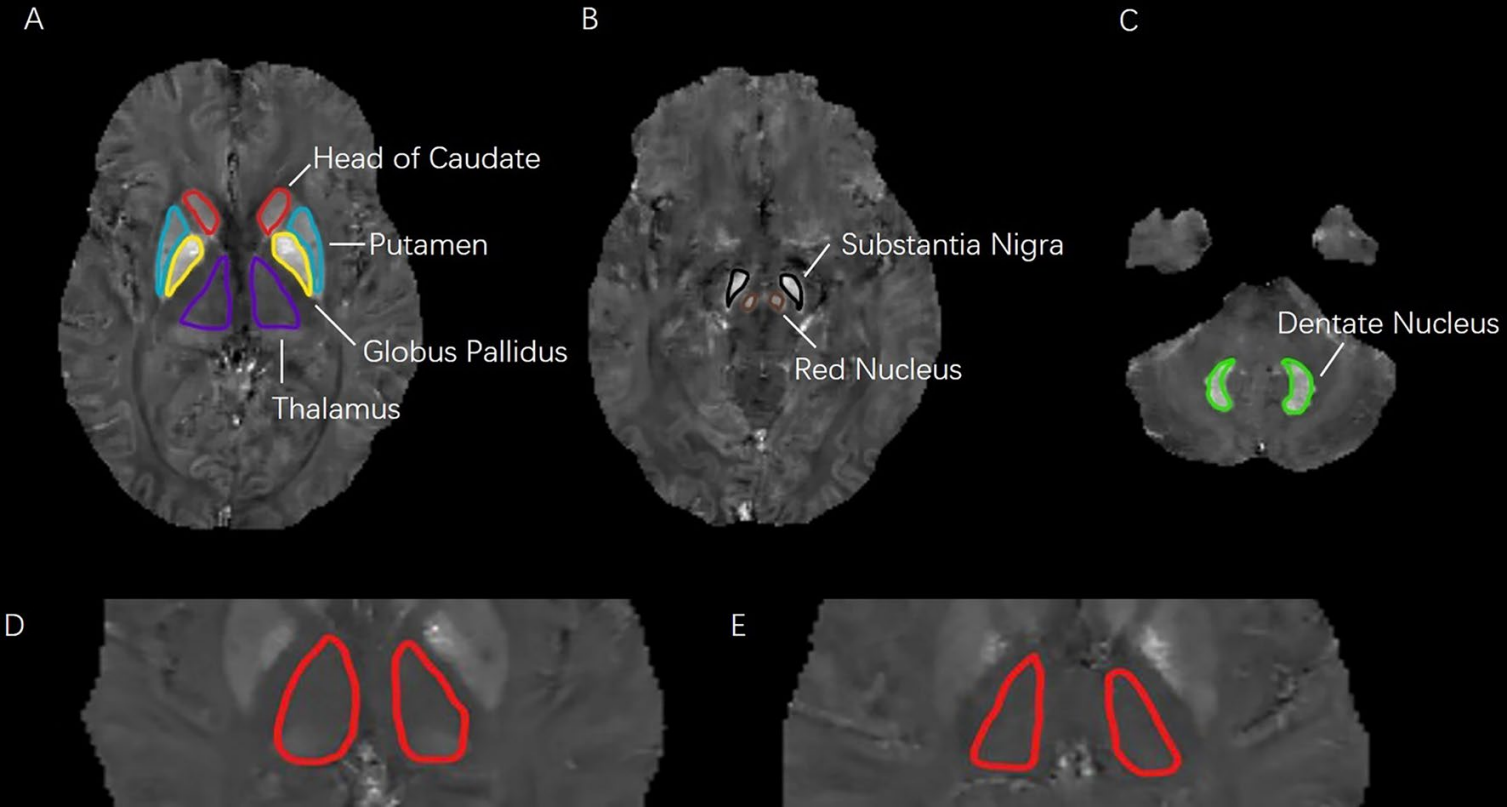
**(A–C)**: Seven regions of interest (ROIs) analyzed in this study. ROIs in three different levels of quantitative susceptibility mapping were including: head of caudate, putamen, globus pallidus, thalamus, substantia nigra, red nucleus, and dentate nucleus. **(D**–**E)**: Difference in QSM images of bilateral thalamus. **(D)**: depressive symptom with HDRS score of 17; **(E)**: health control with HDRS score of 3.

According to the published methods (14), the QSM reconstruction was achieved through the use of a C++ software developed and validated by Wang and his colleagues (15), and is based on nonlinear morphology-enabled dipole inversion (MEDI), which makes use of the consistency between the susceptibility maps and magnitude images obtained from the Spoiled Gradient Recalled Echo (SPGR) acquisitions. The susceptibility maps are obtained through estimating (nonlinearly) the phase maps, which have to be unwrapped and subsequently undergo dipole inversion. The regularization parameter has been fixed and set to 1000. The regions of interest (ROIs) were manually drawn on QSM maps by two experienced neuro-radiologists (YZ and QL) who were blinded to all other imaging and clinical data. ROIs were put on the slices where the boundaries of target nuclei could be seen most clearly. Susceptibility values were averaged within each ROI from three successive slices. Both left and right sides of the target nuclei were measured, and the average values were calculated based on the volume. Globus pallidus, head of caudate nucleus, putamen, red nucleus, substantia nigra, thalamus, and dentate nucleus were contained in the ROIs (**Figure 1**). The segmentation function of spm12 in MATLAB (R2014a) was used to get the ROIs of white matter and gray matter. Absolute QSM values of the ROIs were measured automatically on mricron software.

### Clinical Assessment

We used the Hamilton Depression Rating Scale (HDRS) to assess depressive symptoms and Mini–Mental State Examination (MMSE) to assess cognitive ability at the same time as the MRI scan. According to the HDRS score, the participants were subdivided into two groups: depressive symptom group (HDRS > 7) and control group (HDRS ≤ 7) (16).

### Statistical Analysis

Since the WMHs volume was skewed towards the left of mean, we performed natural log transformations of WMHs volume before the correlation analysis. The log-transformed WMHs volume appeared to be acceptably normative. Independent samples’ two-tailed t-test was used to compare the demographics, vascular factors, HDRS score and imaging data between depressive symptom group and control subjects. Fisher’s Exact test was used for categorical data. We also conducted logistic regression analysis to provide an odds ratio statistic to facilitate comparison with other known risk factors. Partial Pearson’s correlation analysis was conducted to determine the correlation among regional QSM values, log-transformed WMHs volume and HDRS scores, by adjusting for baseline sociodemographic and vascular risk factors. Statistical significance was set at a probability value of < 0.05. All statistical analysis was performed with SPSS 17.0 (SPSS Inc., Chicago, USA).

## Results

### Subject Characteristics

185 consecutive participants were enrolled in this study, after 12 participants were excluded due to poor image quality and 2 participants were excluded due to the history of psychotropic drug use. **Table 1** shows the sociodemographic characteristics, vascular risk factors, volume of WMHs and regional QSM values.

**Table 1:** Sociodemographic characteristics, vascular risk factors, white matter hyperintensities (WMHs) volume and regional quantitative susceptibility mapping (QSM) values in all included participants.

	Value (n (%) or mean)
Sociodemographic Characters	
Female	92 (49.7%)
Age, year	59.70 ± 7.15
Year of education, year	8.31 ± 4.69
MMSE	25.17 ± 5.66
HDRS	2.58 ± 4.28
Vascular Risk Factors	
Hypertension	66 (35.7%)
Diabetes mellitus	20 (10.8%)
Hyperlipidemia	22 (11.9%)
Imaging Features	
Volume of WMHs, ml	6.26 ± 9.23
Volume of DWMHs, ml	1.93 ± 3.72
Volume of PVHs, ml	4.33 ± 6.04
QSM value	
Red Nucleus, ×10^-3^ ppm	118.68 ± 43.08
Substantia Nigra, ×10^-3^ ppm	151.77 ± 54.29
Globus Pallidus, ×10^-3^ ppm	197.96 ± 74.39
Putamen, ×10^-3^ ppm	86.79 ± 27.36
Head of Caudate, ×10^-3^ ppm	77.26 ± 21.83
Thalamus, ×10^-3^ ppm	14.04 ± 9.03
Dentate Nucleus, ×10^-3^ ppm	89.56 ± 47.80

MMSE, Mini–Mental State Examination; HDRS, Hamilton Depression Rating Scale

DWMHs, deep white matter hyperintensities; PVHs, periventricular white matter hyperintensities.

### Reliability of the QSM Value Measurements

The intraclass correlation coefficients (ICCs) were 0.88 for red nucleus, 0.99 for substantia nigra, 0.87 for globus pallidus, 0.96 for putamen, 0.91 for head of caudate, 0.97 for thalamus and 0.97 for dentate nucleus. ICCs were described in detail elsewhere (17).

### Proof of QSM Data

**Figure 2:**
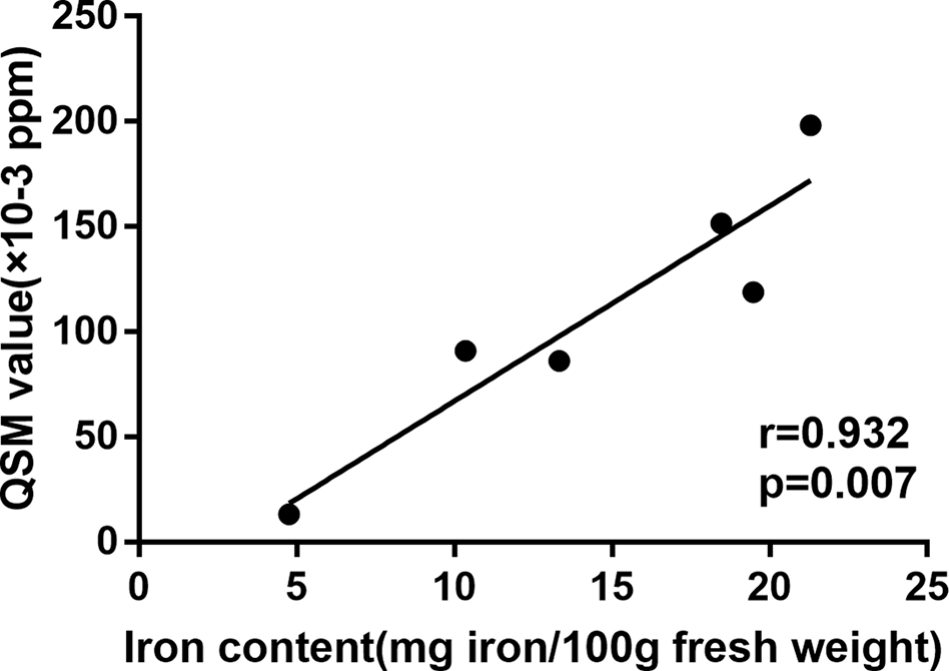
Correlation between the mean QSM value of deep intracranial nuclei from the control group and the mean iron distribution in postmortem samples as reported by Hallgren and Sourande.

Pearson’s correlation analysis was conducted to determine the correlation between the mean QSM value of deep intracranial nuclei from the control group in the present study and the mean iron distribution in postmortem samples as reported by Hallgren and Sourander in 1958 (18), which measured the content of iron in different brain regions and found the relationship between increased age and increased iron deposition. Globus pallidus, red nucleus, substantia nigra, putamen, dentate nucleus, and thalamus were selected as reference region for comparison. A significant correlation (r = 0.932, *p* = 0.007; **Figure 2**) was found and it supported that the QSM data provide a quantitative measure of iron.

### Comparison of Iron Deposits and WMHS Volume Between Depressive Symptom Group and Control Group

**Table 2** shows the sociodemographic characteristics, vascular risk factors, volume of WMHs and regional QSM values of depressive symptom group and control group. The HDRS scores of the depressive symptom group were significantly higher than the control group (11.43 ± 5.32 vs 1.32 ± 2.06, *p* < 0.001). There were no differences between two groups in gender, age or vascular risk factors. Participants in depressive symptom group had lower years of education (6.29 ± 4.42 vs 8.64 ± 4.69, *p* = 0.022), lower MMSE score (22.83 ± 4.38 vs 25.53 ± 5.73, *p* = 0.028), higher volume of total WMHs (10.87 ± 18.65 vs 5.54 ± 6.63, *p* = 0.006), higher volume of PVHs (7.18 ± 11.70 vs 3.93 ± 4.65, p = 0.015), higher volume of DWMHs (4.03 ± 7.66 vs 1.63 ± 2.65, p = 0.003) and higher QSM value in thalamus (18.79 ± 14.94 vs 13.29 ± 7.64, *p* = 0.003; **Figure 1**) than the control group. No differences were observed in other deep nuclei that had been measured. No difference was observed in total grey matter (376.56 ± 21.96 vs 375.22 ± 28.48, *p* > 0.05) or white matter (176.42 ± 27.10 vs 170.66 ± 27.08, *p* > 0.05), either.

**Table 2:** Comparison of sociodemographic characters, vascular risk factors, white matter hyperintensities (WMHs) volume, and regional quantitative susceptibility mapping (QSM) values between depressive symptom group and control group.

	Depressive symptom group (n = 23)	Control group (n = 162)	P Value
Sociodemographic Characters			
Female (%)	13(56.5%)	79 (48.8%)	0.513
Age, year, mean ± SD	61.96 ± 7.88	59.38 ± 7.01	0.106
Years of education, year, mean ± SD	6.35 ± 4.51	8.59 ± 4.67	0.031
MMSE, mean ± SD	22.74 ± 4.45	25.51 ± 5.74	0.028
HDRS, mean ± SD	11.43 ± 5.32	1.32 ± 2.06	0.000
Vascular Risk Factors			
Hypertension (%)	10 (43.8%)	56 (34.6%)	0.362
Diabetes mellitus (%)	2 (8.7%)	18 (11.1%)	1.000
Hyperlipidemia (%)	3(13.0%)	19 (11.7%)	0.506
Imaging Features			
Volume of WMHs, ml, mean ± SD	11.21 ± 18.99	5.55 ± 6.65	0.006
Volume of DWMHs, ml	4.03 ± 7.66	1.63 ± 2.65	0.003
Volume of PVHs, ml	7.18 ± 11.70	3.93 ± 4.65	0.015
QSM value			
Red Nucleus, ×10^-3^ ppm, mean ± SD	117.17 ± 34.10	118.89 ± 44.30	0.858
Substantia Nigra, ×10^-3^ ppm, mean ± SD	153.05 ± 66.35	151.59 ± 52.60	0.905
Globus Pallidus, ×10^-3^ ppm, mean ± SD	195.73 ± 69.51	198.28 ± 75.25	0.878
Putamen, ×10^-3^ ppm, mean ± SD	91.52 ± 30.61	86.12 ± 26.90	0.377
Head of Caudate, ×10^-3^ ppm, mean ± SD	75.74 ± 18.46	77.48 ± 22.31	0.722
Thalamus, ×10^-3^ ppm, mean ± SD	19.19 ± 14.85	13.31 ± 7.66	0.003
Dentate Nucleus, ×10^-3^ ppm, mean ± SD	80.29 ± 45.19	90.88 ± 48.14	0.321
Grey Matter	376.12 ± 21.96	375.38 ± 28.48	0.906
White Matter	176.42 ± 27.10	170.66 ± 27.08	0.341

MMSE, Mini–Mental State Examination; HDRS, Hamilton Depression Rating Scale

DWMHs, deep white matter hyperintensities; PVHs, periventricular white matter hyperintensities

The binary logistic regression model revealed that the QSM value in thalamus was an independent factor for the presence of depressive symptoms (OR = 1.052; 95% CI: 1.010-1.096; *p* = 0.015) after adjusting for years of education and MMSE score. Furthermore, the QSM value in thalamus was still an independent factor for depressive symptoms (OR = 1.055; 95% CI: 1.011-1.100; *p* = 0.013), after adjusting for years of education, MMSE score and the volume of WMHs. In addition, volume of total WMHs, volume of PVHs, or volume of DWMHs were not influencing factors for depressive symptoms after adjusting for years of education and MMSE score (all *p* > 0.05).

### Correlation Analysis Between Iron Deposits, WMHS Volume and HDRS Score in Depressive Symptom Group

As presented in **Table 3**, in the depressive symptom group, none of the QSM values were significantly correlated with HDRS score (all *p* > 0.05), after adjusting for age, gender, year of education, MMSE score and baseline vascular risk factors. Further adjusting for the volume of WMHs did not change the results (all *p* > 0.05).

**Table 3:** Associations between regional quantitative susceptibility mapping (QSM) values, white matter hyperintensities (WMHs) volume HDRS and Hamilton depression scale (HDRS) score in depressive symptom group.

Brain region	HDRS score		log-transformed WMHs volume
	Partial pearson correlation (model1, r(p))	Partial pearson correlation (model2, r(p))	
Red Nucleus	–0.210 (0.436)	–0.180 (0.522)	–0.232 (0.388)
Substantia Nigra	0.026 (0.923)	0.030(0.917)	–0.019 (0.946)
Globus Pallidus	0.082 (0.762)	0.094 (0.738)	–0.066 (0.808)
Putamen	0.477 (0.062)	0.511 (0.052)	–0.140 (0.606)
Head of Caudate	–0.223 (0.407)	–0.212 (0.448)	–0.090 (0.740)
Thalamus	0.094 (0.730)	0.083 (0.769)	0.077 (0.776)
Dentate Nucleus	–0.097 (0.720)	–0.084 (0.765)	–0.090 (0.740)
log-transformed WMHs volume	0.160 (0.553)	/	/

Model 1 adjusted for age, sex, years of education, MMSE scores and vascular risk factors (hypertension, diabetes, hyperlipidemia); Model 2 adjusted for age, sex, years of education, MMSE scores, vascular risk factors (hypertension, diabetes, hyperlipidemia) and log-transformed WMHs volume; age, sex, years of education, MMSE scores and vascular risk factors (hypertension, diabetes, hyperlipidemia, smoke) were also adjusted for analyzing the association between regional QSM value and log-transformed WMHs volume.

In addition, in the depressive symptom group, no significant correlation was observed between log-transformed WMHs volume and HDRS score (all *p* > 0.05), and no significant correlations were observed between log-transformed WMHs volume and regional QSM value (all *p* > 0.05).

## Discussion

Our main findings include: (1) in older adults, iron deposits in the thalamus was an independent factor for depressive symptoms, even after adjusting for WMHs volume; (2) the severity of iron deposits is not related to severity of depression; (3) WMHs volume was not associated with presence of depressive symptoms or brain iron deposits.

In general, the regional QSM value in our study was relatively low, almost the same as the QSM value of healthy older adults in the previous study (the QSM value of Thalamus: our study vs Bettes et al.’s study 0.014 ± 0.009 vs 0.021 ± 0.0008 ppm) (19).

Previous studies have demonstrated that iron deposits in the thalamus were related to the degree of depression among depression patients and post-stroke patients (7, 20). Our study further confirmed that even in older adults, iron deposits in the thalamus were related to depressive symptoms. Although the pathogenesis of depression is still not sufficiently clear, iron deposits might lead to depressive symptoms by multiple mechanisms.

It has been reported that there were abnormal connections in the thalamus-temporal lobes and thalamus-cortex areas in patients with depression (21). Meanwhile, studies have shown that excessive iron could affect functional connectivity (22). Therefore, we speculate that thalamic iron deposits might cause local neuron and neurotransmitter dysfunction, which affects functional connections, finally leading to depressive symptoms.

In addition, the monoamine hypothesis might mediate the relationship between iron deposits and depression. The lack of monoamines, including serotonin, dopamine, norepinephrine, and epinephrine, would lead to depression. Moreover, deficiency of a certain neurotransmitter could lead to a certain depressive symptom (23). Studies have reported that brain iron deposits could affect monoamine function (24). Therefore, brain iron deposits might reduce the activity of monoamines, leading to the occurrence of depressive symptoms.

Furthermore, some studies suggested that inflammation and oxidative stress also could influence depression (25). Cytokines and other pro-inflammatory mediators were involved in the pathophysiological process of mood regulation, such as neurotransmitter metabolism, neuroendocrine function, anterior cingulate cortical activity, and synaptic plasticity (26). Indeed, animal experiments have proven depression-like behaviors could be induced by brain iron overload through apoptosis pathways among adult rats (27). Moreover, Dixon et al. raised the notion of ferroptosis in 2012, which refers to a form of regulated cell death characterized by the iron-dependent accumulation. The accumulation of intracellular iron would induce accumulation of lipid reactive oxygen species (ROS) and the over-accumulation of lipid ROS results in oxidative stress which finally leads to lethal levels for cell deaths (28). Therefore, iron deposits may also cause depressive symptoms by causing an inflammatory reaction and apoptosis.

Yao et al. (7) identified susceptibility values were higher in putamen of patients with major depressive disorder. Whereas, we did not find a correlation between the iron deposits in putamen and depressive symptoms. In addition, our QSM values of putamen met the range of healthy older adults reported before (19, 29). Therefore, given the differences in the study population, we suspect that the putamen might affect different stages of depression. This hypothesis needs to be confirmed in future.

We did not find a relationship between iron deposits and the severity of depression in the depressive symptom group. The mild symptoms of our study population might explain it. We conducted research in old healthy community populations. Although some of their HDRS scores were greater than 7 points, it could only be considered as depressive symptoms rather than depression. In addition, a previous study which found that iron deposits in thalamus were associated with depression severity did not consider cognitive ability, and the cognitive decline in older adults was likely to affect depression scores.

Surprisingly, we did not find the relationship between volume of WMHs and depressive symptoms after adjusting for years of education and MMSE score. According to the vascular depression hypothesis, WMHs may result in mechanistic disconnection and hypoperfusion, which links cerebrovascular diseases with the depression (8). The heterogeneity of the research population might explain the contradiction between our findings and previous research results. Our included population was not diagnosed with depression but was considered to have depressive symptoms based on their HDRS scores. Even in the depressive symptoms group, the HDRS scores were not high (average HDRS score was 11.38). The damage of white matter in our participants might be too slight to affect the depressive symptoms.

We also found no correlation between WMHs and iron deposits. It might also be explained by the difference of research population. The previous positive finding about the relationship between iron deposits and volume of WMHs was based on a population of in-hospital patients, who had severe white matter damage (average volume of WMHs was 35.95 ml) (9), while our current study included a community population with an average WMHs volume of only 6.22 ml. Pathological studies have demonstrated that myelin has a strong ability to store iron without causing damage (30), which may indicate that the damage of iron deposits to myelin is a late manifestation. Moreover, iron deposits might be an early stage of neurodegeneration and might not produce WMHs (10). One possible but hypothetical scenario would be that the early impact of iron deposits on depressive symptoms might be caused by abnormal functional connection, monoamine dysfunction and inflammation reaction, while during the advanced period, iron deposition would aggravate white matter damage and accelerate emotional disorders.

Our study had strength in its methodology. QSM was regarded as a more accurate way to measure iron deposits because it could avoid the loss of signals and the influence of calcifications when measured by R2* (31). We innovatively analyzed the relationship in older adults as there was only one study in the past that examined the relationship between iron deposits and depression by QSM among patients diagnosed as depression. In addition, we excluded the effects of WMHs and cognitive ability, ensuring that the effect of iron deposits on depressive symptoms was an independent process.

Our study had limitations. First, while the total size is close to 200 patients, the group with depressive symptoms is small which can impact the robustness of the results. Second, it was a cross-sectional study that could only analyze the correlation among depression, WMHs and brain iron, and cannot analyze the cause and effect. Further longitudinal study is needed to clarify this. Third, we did not carry out laboratory tests. Therefore, the health status of the enrolled population has not been fully checked, and some factors that may influence emotional behaviors could be ignored. Fourth, voxel-based QSM analysis might further prompt the phenomenon and mechanism, and further study is needed to clarify this. Fifth, the QSM values were measured by manual delineation. Although the consistency was good, there were still measurement errors.

In summary, our finding indicated that in a community population, thalamic iron deposits were an independent factor for depressive symptoms, but WMHs volumes were irrelevant to either increased iron deposits or depressive symptoms. Our results may help to investigate the underlying pathophysiological mechanism of depression in the future studies. It’s worthy to explore the relationship between depressive and specific parts of the thalamus in future.

## Ethics Statement

All subjects had given written informed consent prior to the study, and the protocol was approved by the local ethics committee. All clinical investigation has been conducted according to the principles expressed in the Declaration of Helsinki.

## Author Contributions

WZ and YZ drafted and revised the manuscript, participated in study concept and design, conducted the statistical analyses, analyzed, and interpreted the data. ML participated in study concept and design, data interpretation and made a major contribution in revising the manuscript. QL, JX, and SY participated in the study design and made contribution in revising the manuscript. YJ and JC assisted in designing the MRI sequences and imaging analysis.

## Funding

This study was supported by National Key Research and Development Program of China (2016YFC1300504), National Natural Science Foundation of China (81622017, 81701150), Science Technology Department of Zhejiang Province (2018C04011), Chinese Cardiovascular Association-V.G Fund (2017-CCA-VG-004), and Basic Public Interests of Research Plan of Zhejiang Province (GF18H090006).

## Conflict of Interest

The authors declare that the research was conducted in the absence of any commercial or financial relationships that could be construed as a potential conflict of interest.
